# Mucosal IgA Responses: Damaged in Established HIV Infection—Yet, Effective Weapon against HIV Transmission

**DOI:** 10.3389/fimmu.2017.01581

**Published:** 2017-11-15

**Authors:** Viraj Kulkarni, Ruth M. Ruprecht

**Affiliations:** ^1^Department of Virology and Immunology, Texas Biomedical Research Institute, San Antonio, TX, United States; ^2^Southwest National Primate Research Center, San Antonio, TX, United States

**Keywords:** human immunodeficiency virus-induced IgA, vaccine-induced anti-HIV IgA, systemic IgA responses, mucosal IgA responses, secretory IgA, immune exclusion of HIV, simian-human immunodeficiency virus, passive immunization with dimeric IgA

## Abstract

HIV infection not only destroys CD4^+^ T cells but also inflicts serious damage to the B-cell compartment, such as lymphadenopathy, destruction of normal B-cell follicle architecture, polyclonal hypergammaglobulinemia, increased apoptosis of B cells, and irreversible loss of memory B-cell responses with advanced HIV disease. Subepithelial B cells and plasma cells are also affected, which results in loss of mucosal IgG and IgA antibodies. This leaves the mucosal barrier vulnerable to bacterial translocation. The ensuing immune activation in mucosal tissues adds fuel to the fire of local HIV replication. We postulate that compromised mucosal antibody defenses also facilitate superinfection of HIV-positive individuals with new HIV strains. This in turn sets the stage for the generation of circulating recombinant forms of HIV. What can the mucosal B-cell compartment contribute to protect a healthy, uninfected host against mucosal HIV transmission? Here, we discuss proof-of-principle studies we have performed using passive mucosal immunization, i.e., topical administration of preformed anti-HIV monoclonal antibodies (mAbs) as IgG1, dimeric IgA1 (dIgA1), and dIgA2 isotypes, alone or in combination. Our data indicate that mucosally applied anti-HIV envelope mAbs can provide potent protection against mucosal transmission of simian-human immunodeficiency virus. Our review also discusses the induction of mucosal antibody defenses by active vaccination and potential strategies to interrupt the vicious cycle of bacterial translocation, immune activation, and stimulation of HIV replication in individuals with damaged mucosal barriers.

## Overview: The AIDS Epidemic and HIV-Induced Damage of Mucosal B Cells

Since the beginning of the HIV/AIDS epidemic, more than 35 million people have died (http://www.who.int/gho/hiv/en/); were it not for the introduction of combination antiretroviral therapy, the number of deaths would surpass those caused by the Black Plague in the fourteenth century and the Spanish Flu in 1918, making HIV the worst newly emerged pandemic in human history. An estimated 90% of all new HIV acquisitions occur through mucosal contact, including sexual and perinatal transmission, in which mucosal fluids and tissues are the first points of contact for HIV. Despite this, inducing protective mucosal immune responses by candidate HIV/AIDS vaccines has not been a major focus for most experimental vaccine approaches. Almost all acute HIV acquisitions involve R5-tropic strains, even when the infected source person harbors predominately dual or X4-tropic HIV strains. As such, prevention of virus acquisition by active and/or passive immunization should focus on blocking mucosal transmission of R5 HIV.

B-cell dysregulation was noted at the very beginning of the HIV/AIDS epidemic, even before the viral etiology of this new syndrome was identified [reviewed in Ref. (1)]. Damage to the B-cell compartment was subsequently described as including lymphadenopathy, loss of normal B-cell follicle architecture in lymph nodes, polyclonal hypergammaglobulinemia, altered expression of homing receptors on the surface of B cells and, therefore, increased turnover of such cells, increased apoptosis of B cells due to activation-induced cell death, and eventually irreversible loss of memory B-cell responses with advancing HIV disease. The latter becomes evident by significant decreases in antiviral antibody titers (1–6).

IgA-producing B cells and plasma cells are not spared from the HIV or SIV-induced damage. Mestecky and colleagues (7, 8) described unusually low anti-HIV IgA responses when compared to IgG responses in mucosal fluids. In this review, we discuss the implications of such B-cell damage in infected individuals. We will contrast these findings with the potential role mucosal IgA can play in protecting uninfected hosts from invading HIV or related primate immunodeficiency viruses. Such protection could be provided by passively administering recombinant anti-HIV antibodies directly into mucosal compartments. Alternatively, vaccine strategies can be designed to induce protective anti-HIV mucosal antibody responses. Our review will summarize relevant data generated in non-human primate (NHP) models.

## Mucosal Antibody Production in Normal Hosts

**Figure 1:**
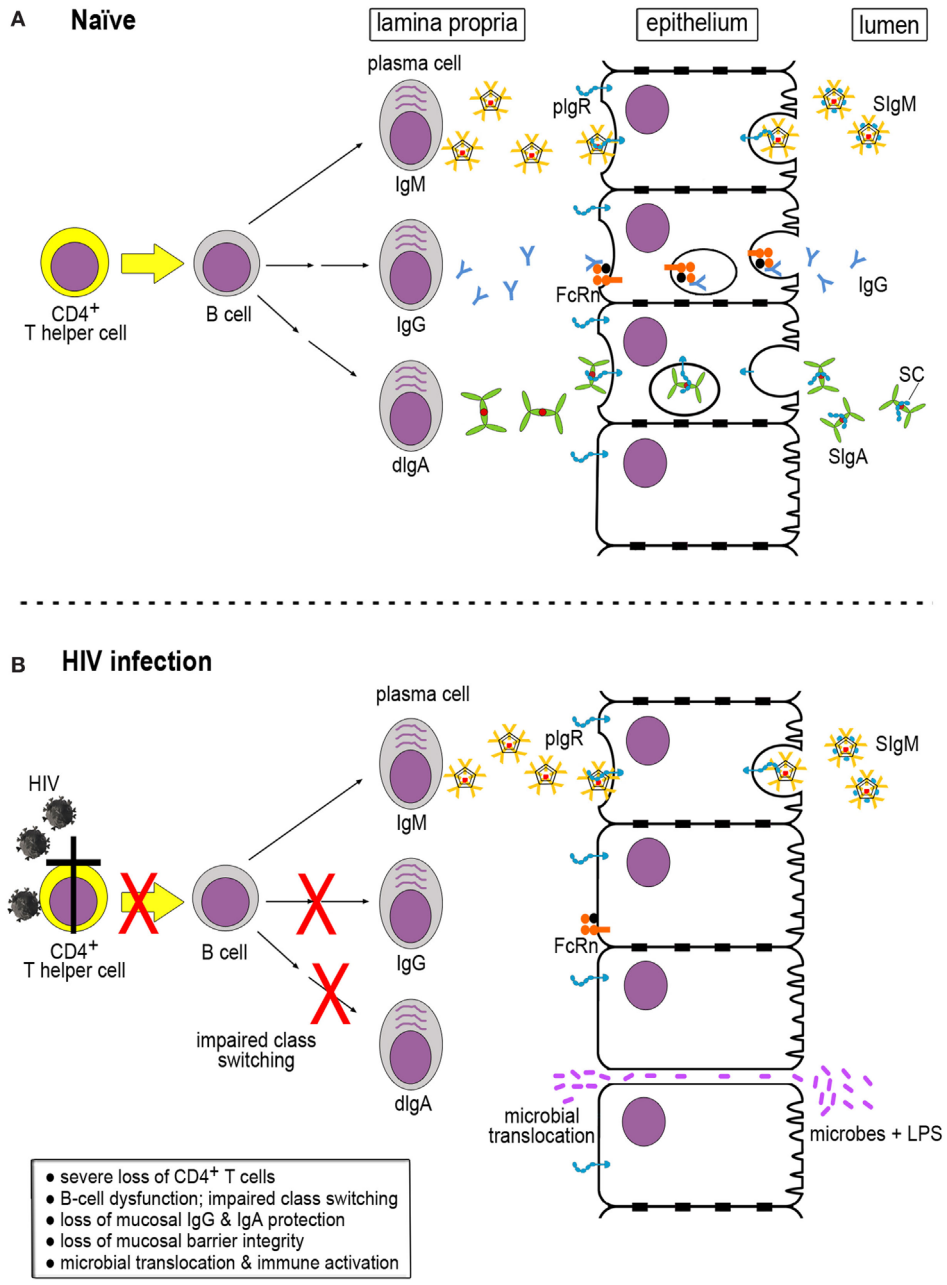
Generation of immunoglobulins (Igs) in naïve and HIV-infected hosts. **(A)** In a naïve host, multimeric IgM, IgG, and dimeric IgA (dIgA) are produced in the lamina propria by mature plasma cells. The latter are derived from B cells that have received help from CD4^+^ T cells for Ig class switching. IgM and dIgA interact with the polymeric immunoglobulin receptor (pIgR), which exports the Igs across the epithelial cells. pIgR is cleaved by proteolysis at the luminal side, resulting in the generation of secretory component (SC) that is retained by IgM and dIgA, giving rise to secretory IgM (SIgM) and IgA (SIgM and SIgA, respectively). IgG binds to the neonatal Fc receptor (FcRn) expressed by epithelial cells that transports IgG across to the luminal side. Unlike pIgR, FcRn does not undergo proteolytic cleavage at the luminal side and can shuttle back and forth. **(B)** During HIV infection, severe loss of CD4^+^ T cells occurs, resulting in impaired Ig class switching and B-cell dysfunction. As a consequence, IgG and SIgA are markedly decreased in the mucosal compartment. Lack of sufficient mucosal barrier defenses leads to loss of barrier integrity, microbial translocation, and immune activation.

In order to understand the dysfunction of the B-cell compartment in HIV infection, it is important to understand the processes involved in generating mucosal antibodies of different classes in healthy, uninfected hosts. Mucosal fluids contain IgM, IgG, and IgA in different forms, especially polymeric versions. These antibodies are produced by local plasma cells in the lamina propria. IgM-producing cells secrete multimeric IgM that contains the joining (J) chain and is generally pentameric. This IgM binds to the polymeric immunoglobulin receptor (pIgR) expressed on the basolateral surface of the epithelial cell barrier. The pIgR–IgM complexes are transported across the epithelial monolayer in transcytotic vesicles and released at the luminal side through a process involving proteolytic cleavage of pIgR. This results in release of the secretory component (SC) that remains associated with IgM, thus generating secretory IgM (Figure 1A, top).

IgG is produced in the bone marrow from where it enters the circulation and is distributed throughout the body tissues. IgG can also be generated locally by subepithelial plasma cells; its trans-epithelial transport occurs through the neonatal Fc receptor (FcRn) (Figure 1A, middle). In contrast to pIgR, FcRn is not degraded upon release of its IgG cargo at the luminal side; this receptor can shuttle back and forth between the luminal and the basolateral aspects of the epithelial cells and carrying IgG-antigen immune complexes from the luminal side across the epithelium into the subepithelial space (Figure 1A, middle) (9).

Like the other immunoglobulin (Ig) classes, IgA destined for mucosal secretions is also produced locally by plasma cells in the lamina propria. B cells release dimeric IgA (dIgA), which consists of two IgA monomers linked at their Fc alpha ends by the J chain. Like IgM, dIgA molecules bind to pIgR at the basolateral aspect of epithelial cells and get transported in transcytotic vesicles to the luminal side, where pIgR undergoes cleavage into a remnant stump and SC, giving rise to secretory IgA (SIgA) (Figure 1A, bottom) (10) found in mucosal fluids. The relative ratios of mucosal IgM:IgG and IgM:IgA vary and depend on the active participation of CD4^+^ T-helper cells, which provide critical stimulatory signals to B cells to undergo Ig class switching.

In humans, IgA exists as closely related subclasses, IgA1 and IgA2, which differ predominantly in the hinge region. In IgA1, the latter contains 19 amino acids (aa) as well as several O-linked oligosaccharides. In contrast, the IgA2 hinge region is only 6 aa long and lacks glycosylation. Due to their open hinge region, IgA1 molecules have a T-like shape with a distance between Fab fragments of approximately 16 nm. Conversely, IgA2 is *Y*-shaped, and the distance between Fab regions measures only 10 nm due to the shorter, stiffer hinge region. The structural differences between IgA1 and IgA2 molecules likely result in different biological activities. Of note, only humans and great apes have the IgA1 subclass with the remarkably open hinge. Rhesus macaques only encode the IgA2-like subclass [reviewed in Ref. (10)].

Among the three classes of mucosal antibodies, IgA in its various forms clearly stands out. The human body generates more IgA per day than all other classes of Igs combined (11), and since IgA ranks only second in the plasma concentration after IgG, it is obvious that the majority of IgA is destined to enter mucosal fluids that need to be replaced continuously. These facts imply a critical role for IgA function in the mucosal compartment.

## Mucosal Antibody Production in HIV/SIV-Infected Hosts

### Skewing of Mucosal Antibody Production in HIV/SIV Infection

Severe depletion of CD4^+^ T cells in the lamina propria and in epithelial tissues during acute SIV infection was first described by Smit-McBride and colleagues (12). This observation was followed by the recognition that the significant loss of the gut-associated lymphoid tissue (GALT) had serious consequences: loss of barrier integrity as demonstrated by increases in plasma concentration of lipopolysaccharide (13, 14). Mattapallil et al. (15) confirmed the loss of GALT CD4^+^ T cells and characterized the affected cell population as memory CD4^+^ T cells.

Among CD4^+^ T cells affected early and severely during HIV/SIV infection is the T helper 17 (Th17) population, a favorite target of primate immunodeficiency viruses. Th17 cells tend to localize preferentially to the gastrointestinal tract where they express a number of genes found to be involved in the maintenance of epithelial cells, including interleukin-22 (IL-22) (16, 17). Favre et al. (18) made an important contribution toward understanding the consequences of severe losses in Th17 cells. These authors compared the acute stage of experimental infection with SIVagm in the setting of a pathogenic host–virus interaction in Asian pig-tailed macaques with that in African green monkeys (AGMs), the natural SIVagm host where the infection remains non-pathogenic. Only pig-tailed macaques but not AGMs suffered immune activation and severe, selective depletion of Th17 cells systemically and in mucosal tissues.

Loss of CD4^+^ T-helper cell function greatly reduces Ig class switching in subepithelial B cells, which results in a significant loss of IgG and SIgA in mucosal fluids (Figure 1B). The serious loss of IgG and dIgA production in the lamina propria leads to a strong skewing of the IgG:IgM and dIgA:IgM ratios, with the IgA content of mucosal fluids in HIV/SIV infection being most severely affected [reviewed in Ref. (19)]. This relative lack of mucosal IgA and IgG results in impaired immune exclusion of bacterial pathogens and makes the epithelial barrier vulnerable to breaches (Figure 1B, bottom). Indeed, during acute SIV as well as HIV infection, bacterial translocation occurs, which results in immune activation and further upregulation of virus replication, starting off a vicious cycle.

### Bacterial Translocation: Adding Fuel to the Fire

Bacterial translocation has serious, deleterious consequences to the host. The most important one is triggering inflammatory responses, resulting in general immune activation. Macrophages, instead of phagocytosing bacteria or bacterial products that may have crossed the epithelial barrier in normal epithelial homeostasis, now send out inflammatory signals that in turn create a more fertile ground for HIV/SIV to spread locally in mucosal tissues [reviewed in Ref. (17)]. Factors involved in this immune activation include tumor necrosis factor-α, which is released from macrophages, and interferon-α (IFN-α), which is produced by plasmacytoid dendritic cells and macrophages. Activated monocytes/macrophages produce soluble CD14 (sCD14) and soluble CD163 (sCD163). High plasma levels of sCD14 were found to be an independent poor prognostic sign for survival of HIV-infected individuals (20).

Mucosal dendritic cells (DCs) play an important role in the local immune activation following bacterial translocation. While DCs are key players in the adaptive immune defenses that benefit the host, these cells also contribute to local immune activation. They release inflammatory cytokines as well as type 1 interferons that damage the Th17 cell population while favoring T regulatory cells in intestinal tissues (18). In addition, DCs can trans-infect CD4^+^ T cells. Such responses greatly intensify local mucosal virus replication [reviewed in Ref. (21)]. Ultimately, bacterial translocation and the ensuing immune activation lead to further damage of mucosal integrity through a vicious cycle of increased virus replication followed by increased loss of CD4 T-helper cell function, which ultimately leaves the mucosal barrier devoid of the protective IgA and IgG antibodies (Figure 1B).

### Compromised Mucosal Antibody Production and HIV Genetic Diversity Worldwide

We hypothesize that loss of epithelial integrity, which leads to bacterial translocation, immune activation, and ultimately to increased numbers of activated HIV target cells, will have another serious consequence: superinfection with new strains of HIV. The compromised local mucosal environment will facilitate transmission of new HIV strains and support high levels of replication of the incoming strain. If the latter infects a cell already harboring the preexisting virus, the two HIV genomes will recombine to generate circulating recombinant forms (CRFs). This is a frequent event in the ongoing HIV pandemic as reflected by the ever increasing complexity of viral genomes with an increasing fraction of CRFs. Inter and intra-clade recombinations are known to occur (https://www.hiv.lanl.gov/content/sequence/HIV/CRFs/CRFs.html) (22–24). In the case of an individual with HIV infection, broad, anti-HIV cell-mediated immune responses encompassing multiple epitopes, which controlled the primary virus, have not prevented superinfection during structured treatment interruption (25).

We propose that the loss of mucosal barrier function is one of the main drivers of the rapidly evolving genetic complexity of HIV during the ongoing pandemic (26). At the entire human population level, superinfection with unrelated HIV strains is problematic. The increasing multitude of genetically evermore divergent strains increases the level of difficulty to find protective HIV vaccines. Superinfection is also deleterious at the level of the superinfected individual, who will experience a second phase of acute viremia. Neutralizing antibodies against the new HIV strains will most likely not exist. If so, high viral loads will ensue and increase immune activation throughout the body. This in turn will increase the damage to the CD4^+^ T-helper cell population and accelerate disease progression. Given the increasing prevalence of CRFs, we hope that our hypothesis will stimulate research on a possible link between loss of mucosal barrier integrity and the prevalence of CRFs. We feel that very early onset of antiretroviral therapy may limit mucosal damage and thus lower the risks of superinfection, which would result in slowing the rate of CRF prevalence. As long as access to antiretroviral drugs remains limited in developing countries, the rates of superinfection may not decline. Finding ways to protect mucosal barriers in already infected individuals and thereby lowering the chances of HIV superinfection could be additional protective mechanisms for individuals with chronic HIV infection.

## Harnessing Mucosal IgA to Protect the Host

The potential role of IgA in providing protection to the host has been controversial. The RV144 phase III efficacy trial performed in Thailand showed a modest but significant lowering of the risks of HIV acquisition among the vaccinees of 31.2%, a result that gave impetus to analyze the immune responses responsible for this effect (27). Surprisingly, neither neutralizing antibodies nor cell-mediated immune responses were linked to the lowering of virus acquisition risks. Rather, serum IgG with antibody-dependent cellular cytotoxicity (ADCC) activity targeting the V1V2 region was associated with beneficial outcome. The latter seemed to be counteracted by serum IgA responses targeting HIV envelope. Mucosal samples were not available for analysis in this trial (28).

### Passive Immunization: A Tool to Show Cause and Effect between Antibodies and Protection

To settle the issue whether anti-HIV Env IgA responses harm or help the host, passive immunization is the tool of choice. This avoids influences of any other immunological principles and restricts the experimental parameters to only the passively administered antibody. This approach is required to dissect the potentially protective roles of anti-HIV humoral responses in the systemic as well as in the mucosal compartments. To our knowledge, passive immunization with systemically administered anti-HIV IgA has not been performed to assess prevention of simian-human immunodeficiency virus (SHIV) acquisition in NHP models. In contrast, we have used passive immunization with topically administered, monoclonal dIgAs to ask whether such monoclonal antibodies (mAbs) could prevent SHIV acquisition after mucosal challenge (29, 30). Of note, passive immunization is the best tool to give cause/effect information between a well-characterized monoclonal antibody and the degree of protection, as no other immunological mechanisms are provided to account for the outcome of the study.

**Figure 2:**
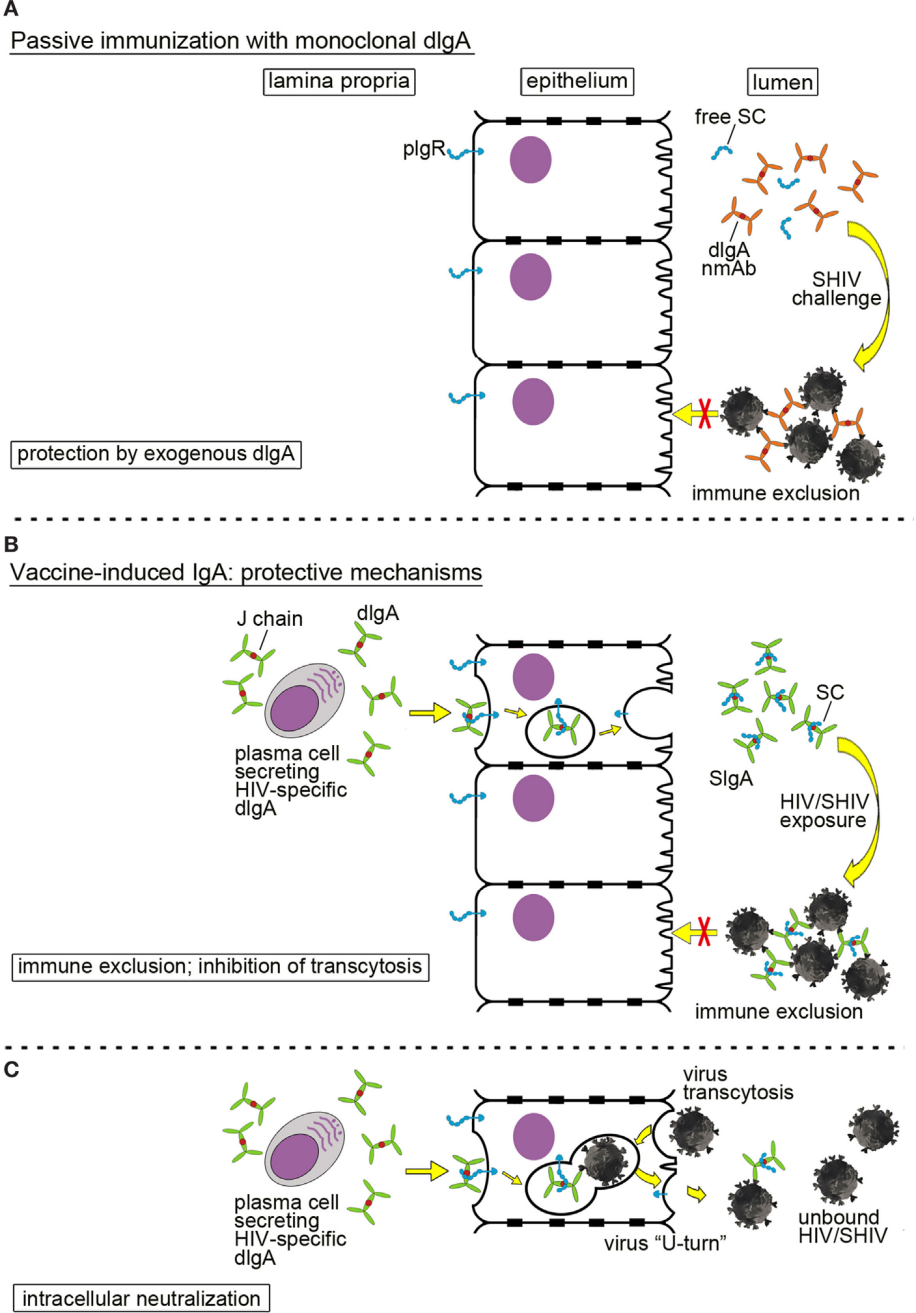
Mucosal defenses by dimeric IgA (dIgA) or secretory IgA (SIgA) against HIV/simian-human immunodeficiency virus (SHIV). **(A)** Passive immunization with a neutralizing monoclonal anti-HIV Env dIgA. Topically administered neutralizing dIgA monoclonal antibodies prevent SHIV from crossing the epithelial barrier by formation of large immune complexes, leading to immune exclusion. To indicate the exogenous source and the recombinant nature of the monoclonal dIgA, the latter are colored in ochre in contrast to dIgAs produced locally by the host (green dIgAs; Figures 1A and 3B,C). **(B,C)** Mucosal SIgA induced by active immunization and different mechanisms of protection; **(B)** immune exclusion and inhibition of transcytosis. Plasma cells in the lamina propria produce virus-specific dIgA that interacts with the polymeric immunoglobulin receptor (pIgR; blue) on the basolateral surface of epithelial cells; pIgR transports dIgA across epithelial cells in transcytotic vesicles. Proteolytic cleavage of pIgR at the luminal side generates secretory component (SC) that is retained by dIgA molecules. The latter complexes are released as SIgA into the lumen. Virion invasion of epithelial cells is blocked by formation of large immune complexes between SIgA and SHIV leading to immune exclusion. **(C)** Intracellular neutralization. This phenomenon occurs when virions are taken up by epithelial cells and enter transcytotic vesicles, in which dIgA-pIgR cargo is being exported toward the lumen. Virions are bound by specific dIgA, and the entire virion-dIgA-pIgR complex is excreted. Essentially, the virion in the complex is forced into a U-turn. This figure has been adapted from the original review article “Are anti-HIV IgAs good guys or bad guys?” by Zhou and Ruprecht (10) (https://retrovirology.biomedcentral.com/articles/10.1186/s12977-014-0109-5). The original article is an open access article distributed under the terms of the Creative Commons Attribution License (http://creativecommons.org/licenses/by/2.0), which permits unrestricted use, distribution, and reproduction in any medium, provided the original work is properly cited.

Passive immunization with mucosally administered, monoclonal dIgAs is depicted in Figure 2A. Any significant prevention of SHIV acquisition would have to occur in the mucosal lumen by trapping infectious virion in large complexes to prevent mucosal transcytosis; this process is called immune exclusion (10, 31). This information needs to be generated in order to assess the role of mucosal B cells and anti-HIV mucosal antibodies in preventing virus acquisition—key data for future vaccine design against a pathogen that is predominantly transmitted *via* mucosal routes.

### Passive Mucosal Immunization with Monoclonal dIgAs

Our group has performed passive mucosal immunization studies with monoclonal dIgAs to test their protective potential against intrarectal SHIV challenge. We generated dIgA1, dIgA2, and IgG1 versions of a neutralizing anti-HIV mAb, HGN194, which targets the conserved V3 loop crown of HIV gp120 (32). This mAb neutralized all tier 1 strains tested and selected tier 2 strains in which the V3 loop crown was accessible. When given intravenously (i.v.) at full dose (50 mg/kg) 24 h prior to high-dose intrarectal challenge with a clade C SHIV (SHIV-C), this mAb provided 100% cross-clade protection to the rhesus macaques (33). Next, we sought to test whether administering the dIgA1, dIgA2, and IgG1 isoforms of HGN194 intrarectally would protect RMs against subsequent intrarectal SHIV-C challenge. All three isoforms neutralized the challenge virus equally well in tissue culture systems. However, the *in vivo* study yielded a surprising result: the dIgA1 isoform was significantly more potent in preventing intrarectal SHIV transmission compared to the dIgA2 form (*p* < 0.05) (29). In this first proof-of-concept study of passive mucosal immunization with recombinant dIgAs, better *in vivo* protection by dIgA1 compared to dIgA2 was linked to better virion capture *in vitro* and inhibition of transcytosis of cell-free virus in a transwell assay (29).

### Protective Mechanisms of IgA in the Mucosal Lumen and Barrier

IgA in mucosal fluids can mediate protection by direct neutralization, immune exclusion, or inhibition of transcytosis. In our passive immunization studies, we have shown that administering of neutralizing dIgA intrarectally prevented SHIV acquisition after intrarectal virus challenge (Figure 2A). The incoming SHIV could either be directly neutralized by dIgAs in the mucosal lumen. Alternatively, large immune complexes could be formed that trap the incoming virus and prevent it from traversing the epithelial barrier *via* transcytosis. Such a mechanism is termed immune exclusion.

IgA responses induced by vaccination can also block HIV/SHIV infection (Figure 2B). The HIV-specific dIgAs produced by subepithelial plasma cells and transported across the epithelial layer into the lumen could mediate protection through immune exclusion or by inhibiting transcytosis. Another interesting mechanism of IgA-mediated protection is intracellular neutralization (Figure 2C). This occurs when a virion is taken up by an epithelial cell and enters the same transcytotic vesicle in which dIgA-pIgR cargo is being carried toward the lumen. Virus is bound by dIgA, forced to make a U-turn, and excreted back into the lumen. This phenomenon was described by Burns et al. (34) for the rotavirus murine model.

### The Interplay between Mucosal dIgAs and IgGs

Mucosal fluids are known to contain not only IgA in the form of SIgA but also IgG. We sought to test whether the dIgA version would interfere with the protection provided by the IgG1 forms—as had been implied by the systemic IgG and IgA antibodies in the RV144 trial. Since the hypothesis was that the IgA form would decrease the protective effect of the IgG, we deliberately selected the dIgA2 version of HGN194, which had given suboptimal protection of only 17% when used as a single agent. In contrast, the dIgA1 version had provided 83% protection. Instead of delivering the IgG mucosally, we decided to administer a suboptimal dose i.v. 24 h before intrarectal SHIV-C challenge to allow the antibody to distribute in tissues and enter mucosal fluids.

We enrolled three groups of RMs; one group was given only the i.v. IgG1form of HGN194 24 h before virus challenge, the second group was given the same IgG treatment and an additional intrarectal passive immunization 30 min before virus challenge. The controls were left untreated. By itself, the low-dose i.v. IgG form gave no protection. The dIgA2 version by itself had given 17% protection in the previous study (29). Surprisingly, the low-dose i.v. IgG1 combined with the intrarectally administered dIgA2 yielded 100% protection (30). This *in vivo* synergy cannot be explained by synergistic neutralization *in vitro*. Rather, we postulate that local interactions with mucins and epithelial barrier structures may underlie this remarkably potent protection. This unexpected result has since been replicated and again yielded 100% protection (unpublished data).

To summarize, passive immunization has revealed a potent protective mechanism at the level of the mucosal barrier that can yield 100% protection by combining mucosal IgG with dIgAs. It will be important to elucidate the mechanisms of this interaction in future studies. It needs to be emphasized also that passive immunization involves only the mAbs administered, in the absence of any other potentially confounding protective mechanisms by the host. These encouraging data provide strong impetus to focus on inducing protective IgG and IgA mucosal antibody responses by targeted active vaccination.

### Induction of Protective Mucosal Antibody Responses by Active Immunization

To date, one vaccine strategy specifically was designed to focus on the induction of mucosal antibody responses: virosomes displaying different fragments of HIV gp41. Virosomes are empty particles derived from influenza virus but devoid of any nucleic acid; as such, this vaccine carrier is noninfectious and has a very good safety profile in clinical studies targeting conditions other than HIV (35, 36). Two populations of virosomes were tested in NHP studies, namely virosome-P1, which displayed the extended P1 peptide mimicking the membrane proximal external region (MPER) of HIV gp41, in a second population of virosomes displaying a truncated form of gp41 lacking the immunodominant mini loop. This second form of virosomes was termed virosome-rgp41. When tested in Chinese-origin rhesus monkeys, 100% of the vaccinated animals were protected from persistent systemic infection when given the combination of the two virosomes by two intramuscular vaccinations followed by two intranasal boosts. This group of vaccinees showed no seroconversion to SIV Gag after multiple low-dose intravaginal challenges with an upfront heterologous R5 tier 2 SHIV, although some of the animals had low level blips of viremia initially (37). These authors performed an extensive analysis to determine the correlates of protection. None of the systemic antibody responses showed any link, including neutralizing antibody responses and systemic ADCC. In contrast, vaginal fluid IgA was linked to protection through inhibition of virus transcytosis in a transwell system and vaginal IgG showed neutralizing and ADCC activity. In other words, only mucosal IgA and IgG but not systemic IgA and IgG responses correlated with protection.

We have independently confirmed these data during the first half of the multiple low-dose vaginal challenges, where we noticed between 78 and 87% protection against the initial challenge virus dose. These repeat studies were conducted in Indian-origin rhesus monkeys. When comparing the virus challenge dose with the viral RNA copy numbers of the average HIV inoculum likely transferred from a HIV positive man to a female partner, the SHIV inoculum used in our study was 70,000 times higher. When we had to increase the SHIV challenge dose in the Indian-origin monkeys in a second part of the virus challenge phase as had been done in the earlier study (37), protection was lost. This virus challenge dose was greater than 100,000 times the average HIV inoculum passed from an infected man to a female partner. We interpret these findings as promising data that warrant optimizing vaccine strategies based upon this platform (unpublished data).

### Are Highly Exposed Persistently Seronegative (HEPS) Individuals Protected by Anti-HIV Mucosal IgA?

A few groups have reported an intriguing link between individuals who despite frequent sexual HIV exposures have remained uninfected—and IgA responses [reviewed in Ref. (10)]. HIV-specific IgA responses have been correlated with resistance to HIV acquisition in sex workers and in persistently uninfected sexual partners of HIV-positive individuals; the methods to isolate mucosal IgA were based upon jacalin resins that preferentially bind to the O-linked oligosaccharides in the wide hinge region of human IgA1 [jacalin specifically binds to IgA1 hinge O-linked oligosaccharides (38–40) reviewed in Ref. (10)]. Epitope mapping revealed that mucosal IgAs targeted relatively conserved MPER epitopes HIV gp41 (41, 42). Mucosal IgAs isolated from HEPS subjects exhibited cross-clade neutralization (43). Other investigators noted that HIV-specific mucosal antibody responses were either not detectable or found in only a low fraction of HEPS in some cohorts (44–47). The disparate findings regarding mucosal IgA isolated from HEPS individual may stem from assay conditions, including the choice of protease inhibitors and the timing of their addition to mucosal fluids, the use of jacalin-based IgA isolation methods that yield predominantly IgA1 isotype antibodies, and assay sensitivity.

More recently, Hirbod et al. (48) described that neutralizing IgA1 in the foreskin of uncircumcised men was associated with lower risks of HIV acquisition. These authors performed blinded analyses on foreskin swabs collected in a randomized Ugandan trial of male circumcision for HIV prevention. The study’s goal was to assess correlates of HIV acquisition risks in foreskin using a case-control design. IgA was isolated by Jacalin column chromatography from swabs, a method that predominately yields IgA1 as mentioned above. The presence of IgA neutralizing capacity in foreskin samples was associated with an odds ratio (OR) of 0.31 for HIV acquisition in these uncircumcised men at initial enrollment and 0.21 at the last visit when cases were still seronegative. These data parallel those obtained in high-risk Kenyan sex workers, where the OR of HIV infection among study subjects with neutralizing IgA in cervical/vaginal secretions was 0.31 (30). Together, data from both studies imply a protective effect of mucosal IgA against sexual HIV transmission.

The presence of neutralizing anti-HIV IgA in the cervico-vaginal secretions of HEPS women in Kenya and Uganda enrolled in the Partners pre-exposure prophylaxis (PrEP) study was confirmed by Lund et al. HEPS women on oral PrEP had significantly higher levels of neutralizing IgA antibodies as compared to placebo controls (49).

In summary, studies on HEPS subjects imply that mucosal anti-HIV IgA responses may be linked to prevention of persistent systemic HIV infection. Understanding the mechanism of protection among these populations will be important in designing effective vaccines.

## Conclusion

The goal of this review was to provide a juxtaposition between the potential of mucosal antibodies in normal hosts to protect against immunodeficiency virus acquisition versus the severely damaged status of mucosal antibody-producing cells in established HIV/SIV/SHIV infections. In uninfected hosts, IgA in mucosal fluids can prevent mucosal virus transmission through a process termed immune exclusion. This was demonstrated in the first proof-of-concept passive mucosal immunization studies involving recombinant monoclonal dIgAs. Thus far, active induction of protective mucosal IgA together with IgG has been achieved only in the vaccine study by Bomsel et al. (37) and by our group (unpublished data).

During the course of natural HIV/SIV/SHIV infection, the production of mucosal antigen-specific IgG and IgA is severely compromised, which leads to a skewing of the IgG:IgM and IgA:IgM ratios in mucosal fluids. It is likely that the low production of mucosal IgA and IgG compromises mucosal barrier integrity. This can lead to microbial translocation that is associated with severe immune activation, an additional mechanism that upregulates virus replication in mucosal tissues. Together, such damages inflicted on mucosal cells, tissues, and barrier function also weaken anti-HIV mucosal antibody responses. This may be a key risk factor in the frequently observed superinfection of HIV-positive individuals, resulting in inter- or intra-clade recombination events and the generation of CRFs. Their ever increasing genetic diversity may be an indirect indicator of loss of mucosal barrier protection due to the damage inflicted upon the mucosal B-cell compartment. Strategies aimed at improving humoral mucosal defenses and prevention of microbial translocation in HIV-infected individuals—perhaps by therapeutic vaccination—may improve the overall health status of individuals with chronic HIV infection.

## Author Contributions

All authors listed have made a substantial, direct, and intellectual contribution to the work and approved it for publication.

## Conflict of Interest Statement

The authors declare that the research was conducted in the absence of any commercial or financial relationships that could be construed as a potential conflict of interest.

## References

[1] Moir S Fauci AS B cells in HIV infection and disease. Nat Rev Immunol (2009) 9(4):235–45. 10.1038/nri2524 19319142 PMC2779527

[2] Zhang ZQ Casimiro DR Schleif WA Chen M Citron M Davies ME Early depletion of proliferating B cells of germinal center in rapidly progressive simian immunodeficiency virus infection. Virology (2007) 361(2):455–64. 10.1016/j.virol.2006.12.006 17223151

[3] Levesque MC Moody MA Hwang KK Marshall DJ Whitesides JF Amos JD Polyclonal B cell differentiation and loss of gastrointestinal tract germinal centers in the earliest stages of HIV-1 infection. PLoS Med (2009) 6(7):e1000107. 10.1371/journal.pmed.1000107 19582166 PMC2702159

[4] Moir S Buckner CM Ho J Wang W Chen J Waldner AJ B cells in early and chronic HIV infection: evidence for preservation of immune function associated with early initiation of antiretroviral therapy. Blood (2010) 116(25):5571–9. 10.1182/blood-2010-05-285528 20837780 PMC3031405

[5] Pensieroso S Galli L Nozza S Ruffin N Castagna A Tambussi G B-cell subset alterations and correlated factors in HIV-1 infection. AIDS (2013) 27(8):1209–17. 10.1097/QAD.0b013e32835edc47 23343911

[6] de Bree GJ Lynch RM. B cells in HIV pathogenesis. Curr Opin Infect Dis (2016) 29(1):23–30. 10.1097/QCO.0000000000000225 26658653

[7] Mestecky J Jackson S Moldoveanu Z Nesbit LR Kulhavy R Prince SJ Paucity of antigen-specific IgA responses in sera and external secretions of HIV-type 1-infected individuals. AIDS Res Hum Retroviruses (2004) 20(9):972–88. 10.1089/aid.2004.20.972 15585085

[8] Mestecky J Moldoveanu Z Smith PD Hel Z Alexander RC. Mucosal immunology of the genital and gastrointestinal tracts and HIV-1 infection. J Reprod Immunol (2009) 83(1–2):196–200. 10.1016/j.jri.2009.07.005 19853927 PMC2802574

[9] Rojas R Apodaca G. Immunoglobulin transport across polarized epithelial cells. Nat Rev Mol Cell Biol (2002) 3(12):944–55. 10.1038/nrm972 12461560

[10] Zhou M Ruprecht RM. Are anti-HIV IgAs good guys or bad guys? Retrovirology (2014) 11:109. 10.1186/s12977-014-0109-5 25499540 PMC4297362

[11] Corthesy B. Multi-faceted functions of secretory IgA at mucosal surfaces. Front Immunol (2013) 4:185. 10.3389/fimmu.2013.00185 23874333 PMC3709412

[12] Smit-McBride Z Mattapallil JJ McChesney M Ferrick D Dandekar S. Gastrointestinal T lymphocytes retain high potential for cytokine responses but have severe CD4(+) T-cell depletion at all stages of simian immunodeficiency virus infection compared to peripheral lymphocytes. J Virol (1998) 72(8):6646–56. 9658111 10.1128/jvi.72.8.6646-6656.1998 PMC109855

[13] Brenchley JM Price DA Schacker TW Asher TE Silvestri G Rao S Microbial translocation is a cause of systemic immune activation in chronic HIV infection. Nat Med (2006) 12(12):1365–71. 10.1038/nm1511 17115046

[14] Douek D. HIV disease progression: immune activation, microbes, and a leaky gut. Top HIV Med (2007) 15(4):114–7. 17720995

[15] Mattapallil JJ Douek DC Hill B Nishimura Y Martin M Roederer M. Massive infection and loss of memory CD4+ T cells in multiple tissues during acute SIV infection. Nature (2005) 434(7037):1093–7. 10.1038/nature03501 15793563

[16] Klatt NR Estes JD Sun X Ortiz AM Barber JS Harris LD Loss of mucosal CD103+ DCs and IL-17+ and IL-22+ lymphocytes is associated with mucosal damage in SIV infection. Mucosal Immunol (2012) 5(6):646–57. 10.1038/mi.2012.38 22643849 PMC3443541

[17] Klatt NR Funderburg NT Brenchley JM. Microbial translocation, immune activation, and HIV disease. Trends Microbiol (2013) 21(1):6–13. 10.1016/j.tim.2012.09.001 23062765 PMC3534808

[18] Favre D Lederer S Kanwar B Ma ZM Proll S Kasakow Z Critical loss of the balance between Th17 and T regulatory cell populations in pathogenic SIV infection. PLoS Pathog (2009) 5(2):e1000295. 10.1371/journal.ppat.1000295 19214220 PMC2635016

[19] Hel Z Xu J Denning WL Helton ES Huijbregts RP Heath SL Dysregulation of systemic and mucosal humoral responses to microbial and food antigens as a factor contributing to microbial translocation and chronic inflammation in HIV-1 infection. PLoS Pathog (2017) 13(1):e1006087. 10.1371/journal.ppat.1006087 28125732 PMC5268400

[20] Sandler NG Wand H Roque A Law M Nason MC Nixon DE Plasma levels of soluble CD14 independently predict mortality in HIV infection. J Infect Dis (2011) 203(6):780–90. 10.1093/infdis/jiq118 21252259 PMC3071127

[21] Manches O Frleta D Bhardwaj N. Dendritic cells in progression and pathology of HIV infection. Trends Immunol (2014) 35(3):114–22. 10.1016/j.it.2013.10.003 24246474 PMC3943663

[22] Grisson RD Chenine AL Yeh LY He J Wood C Bhat GJ Infectious molecular clone of a recently transmitted pediatric human immunodeficiency virus clade C isolate from Africa: evidence of intraclade recombination. J Virol (2004) 78(24):14066–9. 10.1128/JVI.78.24.14066-14069.2004 15564517 PMC533957

[23] Zhang M Foley B Schultz AK Macke JP Bulla I Stanke M The role of recombination in the emergence of a complex and dynamic HIV epidemic. Retrovirology (2010) 7:25. 10.1186/1742-4690-7-25 20331894 PMC2855530

[24] Tongo M Dorfman JR Martin DP. High degree of HIV-1 group M (HIV-1M) genetic diversity within circulating recombinant forms: insight into the early events of HIV-1M evolution. J Virol (2015) 90(5):2221–9. 10.1128/jvi.02302-15 26656688 PMC4810712

[25] Altfeld M Allen TM Yu XG Johnston MN Agrawal D Korber BT HIV-1 superinfection despite broad CD8+ T-cell responses containing replication of the primary virus. Nature (2002) 420(6914):434–9. 10.1038/nature01200 12459786

[26] McCutchan FE Understanding the genetic diversity of HIV-1. AIDS (2000) 14(Suppl 3):S31–44. 11086847

[27] Rerks-Ngarm S Pitisuttithum P Nitayaphan S Kaewkungwal J Chiu J Paris R Vaccination with ALVAC and AIDSVAX to prevent HIV-1 infection in Thailand. N Engl J Med (2009) 361(23):2209–20. 10.1056/NEJMoa0908492 19843557

[28] Haynes BF Gilbert PB McElrath MJ Zolla-Pazner S Tomaras GD Alam SM Immune-correlates analysis of an HIV-1 vaccine efficacy trial. N Engl J Med (2012) 366(14):1275–86. 10.1056/NEJMoa1113425 22475592 PMC3371689

[29] Watkins JD Sholukh AM Mukhtar MM Siddappa NB Lakhashe SK Kim M Anti-HIV IgA isotypes: differential virion capture and inhibition of transcytosis are linked to prevention of mucosal R5 SHIV transmission. AIDS (2013) 27(9):F13–20. 10.1097/QAD.0b013e328360eac6 23775002 PMC4084966

[30] Sholukh AM Watkins JD Vyas HK Gupta S Lakhashe SK Thorat S Defense-in-depth by mucosally administered anti-HIV dimeric IgA2 and systemic IgG1 mAbs: complete protection of rhesus monkeys from mucosal SHIV challenge. Vaccine (2015) 33(17):2086–95. 10.1016/j.vaccine.2015.02.020 25769884 PMC4411954

[31] Ruprecht RM Lakhashe SK Antibody-mediated immune exclusion of HIV. Curr Opin HIV AIDS (2017) 12(3):222–8. 10.1097/COH.0000000000000369 28422786 PMC5604883

[32] Corti D Langedijk JP Hinz A Seaman MS Vanzetta F Fernandez-Rodriguez BM Analysis of memory B cell responses and isolation of novel monoclonal antibodies with neutralizing breadth from HIV-1-infected individuals. PLoS One (2010) 5(1):e8805 10.1371/journal.pone.0008805 20098712 PMC2808385

[33] Watkins JD Siddappa NB Lakhashe SK Humbert M Sholukh A Hemashettar G An anti-HIV-1 V3 loop antibody fully protects cross-clade and elicits T-cell immunity in macaques mucosally challenged with an R5 clade C SHIV. PLoS One (2011) 6(3):e18207. 10.1371/journal.pone.0018207 21483815 PMC3069056

[34] Burns JW Siadat-Pajouh M Krishnaney AA Greenberg HB. Protective effect of rotavirus VP6-specific IgA monoclonal antibodies that lack neutralizing activity. Science (1996) 272(5258):104–7. 10.1126/science.272.5258.104 8600516

[35] Moser C Amacker M Kammer AR Rasi S Westerfeld N Zurbriggen R. Influenza virosomes as a combined vaccine carrier and adjuvant system for prophylactic and therapeutic immunizations. Expert Rev Vaccines (2007) 6(5):711–21. 10.1586/14760584.6.5.711 17931152

[36] Herzog C Hartmann K Kunzi V Kursteiner O Mischler R Lazar H Eleven years of inflexal V-a virosomal adjuvanted influenza vaccine. Vaccine (2009) 27(33):4381–7. 10.1016/j.vaccine.2009.05.029 19450630

[37] Bomsel M Tudor D Drillet AS Alfsen A Ganor Y Roger MG Immunization with HIV-1 gp41 subunit virosomes induces mucosal antibodies protecting nonhuman primates against vaginal SHIV challenges. Immunity (2011) 34(2):269–80. 10.1016/j.immuni.2011.01.015 21315623

[38] Gregory RL Rundegren J Arnold RR. Separation of human IgA1 and IgA2 using jacalin-agarose chromatography. J Immunol Methods (1987) 99(1):101–6. 10.1016/0022-1759(87)90037-8 3106500

[39] Aucouturier P Duarte F Mihaesco E Pineau N Preud’homme JL. Jacalin, the human IgA1 and IgD precipitating lectin, also binds IgA2 of both allotypes. J Immunol Methods (1988) 113(2):185–91. 10.1016/0022-1759(88)90331-6 3171189

[40] Loomes LM Stewart WW Mazengera RL Senior BW Kerr MA. Purification and characterization of human immunoglobulin IgA1 and IgA2 isotypes from serum. J Immunol Methods (1991) 141(2):209–18. 10.1016/0022-1759(91)90147-8 1880427

[41] Pastori C Barassi C Piconi S Longhi R Villa ML Siccardi AG HIV neutralizing IgA in exposed seronegative subjects recognise an epitope within the gp41 coiled-coil pocket. J Biol Regul Homeost Agents (2000) 14(1):15–21. 10763886

[42] Clerici M Barassi C Devito C Pastori C Piconi S Trabattoni D Serum IgA of HIV-exposed uninfected individuals inhibit HIV through recognition of a region within the alpha-helix of gp41. AIDS (2002) 16(13):1731–41. 10.1097/00002030-200209060-00004 12218383

[43] Devito C Hinkula J Kaul R Kimani J Kiama P Lopalco L Cross-clade HIV-1-specific neutralizing IgA in mucosal and systemic compartments of HIV-1-exposed, persistently seronegative subjects. J Acquir Immune Defic Syndr (2002) 30(4):413–20. 10.1097/00042560-200208010-00007 12138348

[44] Dorrell L Hessell AJ Wang M Whittle H Sabally S Rowland-Jones S Absence of specific mucosal antibody responses in HIV-exposed uninfected sex workers from the Gambia. AIDS (2000) 14(9):1117–22. 10.1097/00002030-200006160-00008 10894275

[45] Ghys PD Belec L Diallo MO Ettiegne-Traore V Becquart P Maurice C Cervicovaginal anti-HIV antibodies in HIV-seronegative female sex workers in Abidjan, Cote d’Ivoire. AIDS (2000) 14(16):2603–8. 10.1097/00002030-200011100-00025 11101074

[46] Buchacz K Parekh BS Padian NS van der Straten A Phillips S Jonte J HIV-specific IgG in cervicovaginal secretions of exposed HIV-uninfected female sexual partners of HIV-infected men. AIDS Res Hum Retroviruses (2001) 17(18):1689–93. 10.1089/08892220152741388 11788020

[47] Skurnick JH Palumbo P DeVico A Shacklett BL Valentine FT Merges M Correlates of nontransmission in US women at high risk of human immunodeficiency virus type 1 infection through sexual exposure. J Infect Dis (2002) 185(4):428–38. 10.1086/338830 11865394 PMC2743095

[48] Hirbod T Kong X Kigozi G Ndyanabo A Serwadda D Prodger JL HIV acquisition is associated with increased antimicrobial peptides and reduced HIV neutralizing IgA in the foreskin prepuce of uncircumcised men. PLoS Pathog (2014) 10(10):e1004416. 10.1371/journal.ppat.1004416 25275513 PMC4183701

[49] Lund JM Broliden K Pyra MN Thomas KK Donnell D Irungu E HIV-1-neutralizing IgA detected in genital secretions of highly HIV-1-exposed seronegative women on oral preexposure prophylaxis. J Virol (2016) 90(21):9855–61. 10.1128/JVI.01482-16 27558421 PMC5068535

